# Role of p16^INK4A^ in Replicative Senescence and DNA Damage-Induced Premature Senescence in p53-Deficient Human Cells

**DOI:** 10.1155/2012/951574

**Published:** 2012-08-13

**Authors:** Razmik Mirzayans, Bonnie Andrais, Gavin Hansen, David Murray

**Affiliations:** Department of Oncology, University of Alberta, Cross Cancer Institute, Edmonton, AB, Canada T6G 1Z2

## Abstract

The p16^INK4A^ (hereafter p16) tumor suppressor is encoded by the *INK4A/ARF* locus which is among the most commonly dysregulated sequences in human cancer. By inhibiting cyclin-dependent kinases, p16 activates the G1-S checkpoint, and this response is often considered to be critical for establishing a senescence-like growth arrest. Not all studies support a universal role for p16 in senescence. Single-cell analysis of noncancerous human fibroblast cultures undergoing senescence as a function of culture age (replicative senescence) has revealed that p16 is not expressed in the majority (>90%) of cells that exhibit features of senescence (e.g., flattened and enlarged morphology coupled with senescence-associated **β**-galactosidase expression), ruling out a requirement for p16 in this process. In addition, ionizing radiation triggers premature senescence in human cancer cell lines that do not express p16. These observations are made with cells that express wild-type p53, a key mediator of the DNA damage response. In this paper, we examine the growing evidence suggesting a negative regulatory relationship between p16 and p53 and discuss recent reports that implicate a role for p16 in replicative senescence and ionizing radiation-induced premature senescence in human cells that lack wild-type p53 function.

## 1. Introduction

Normal somatic human cells in culture undergo a finite number of divisions before entering a state of irreversible growth arrest termed ‘‘replicative senescence” [1]. This phenotype is characterized by the acquisition of flattened and enlarged cell morphology, presence of *β*-galactosidase activity at suboptimal conditions (i.e., pH 6), and absence of cell division in metabolically active cells. Replicative senescence is triggered by erosion and dysfunction of telomeres and is mediated by multibranched signaling processes [2, 3]. Exposure of certain immortalized cell types (e.g., p53-proficient human solid tumor-derived cell lines), as well as “young” (early-passage) normal human cells (e.g., skin fibroblasts) to DNA-damaging agents can also trigger a state of sustained growth arrest resembling senescence. The DNA damage-triggered response is commonly called “stress-induced premature senescence” (SIPS). Unlike replicative senescence, SIPS is independent of telomere length or function [3].

Bypassing replicative senescence is a prerequisite step in immortalization and malignant transformation [4], and escape from SIPS can lead to the emergence of highly metastatic and therapy-resistant cells [5, 6]. Accordingly, a great deal of research has been directed towards understanding the molecular basis for different forms of senescence in an attempt to identify novel targets for the treatment of pre-neoplastic lesions and malignant disease.

Ectopic expression of numerous cancer-associated cell-cycle genes (e.g., *p21^WAF1^, p16^INK4A^, p27^KIP1^, p15^INK4B^*, *pRB,* and *CHK2*) in human cells has been reported to trigger senescence (reviewed in [7]). In the absence of artificial gene manipulation, upregulation of the cyclin-dependent kinase (CDK) inhibitors p16^INK4A^ and p21^WAF1^ (hereafter called p16 and p21, resp.) has also been consistently reported to be associated with senescence [8–13]. While the pivotal role of p21 in orchestrating replicative senescence and DNA damage-induced SIPS has been well established [6], attempts to elucidate a role for p16 in these processes have led to inconsistent outcomes, with some reports providing strong evidence for p16-driven senescence (e.g., in human fibroblasts [8] and melanocytes [13] undergoing telomere-directed senescence), and other reports demonstrating the induction of senescence in the absence of p16 (e.g., in human fibroblasts [2] and endothelial cells [14], also undergoing telomere-directed senescence).

Although p16 has been extensively characterized for its ability to decelerate cell progression from G1 to S phase, it has emerged as a multifunctional protein capable of forming a negative regulatory loop with p53, a key mediator of the DNA damage response. In addition, recent work with noncancerous human fibroblast strains and solid tumor-derived cell lines with differing p53 status has implicated the involvement of p16 in a redundant pathway for senescence, triggering this response only in the absence of wild-type p53 activity. Here, we will consider the evidence for these properties of p16.

## 2. Regulation of p16 Expression

The human *INK4/ARF* locus is located on chromosome 9p21 and generates p16 and at least two other transcriptional variants, p14^ARF^ (alternative reading frame) and p12 [15, 16]. Regulation of this locus is complex, involving several tumor-relevant and/or stress signaling pathways [17]. The p38 mitogen-activated protein kinase (MAPK) pathway mediates *p16^INK4A^* induction, the RNA binding protein AUF1 negatively regulates *p16^INK4A^* mRNA stability [17], and the T box proteins (e.g., Tbx2) [18], the polycomb group proteins (e.g., BMI-1) [19, 20], histone deacetylases [21, 22], and the transcription regulators E2F1 and c-MYC [21, 23] repress *p16^INK4A^* expression.

In 2005, Jacobs and de Lange proposed that the p53 tumor suppressor might also contribute to p16 regulation [17]. This notion was based on the observation that upregulation of p16 following DNA damage was unexpectedly delayed, occurring after the initial increase of p53 and its subsequent decline to background levels. That p16 is a target of p53-mediated repression is now well documented. Hernández-Vargas et al. [24], for example, reported that p53 transcriptionally activates the helix-loop-helix transcriptional regulator protein Id1, a well-known repressor of *p16^INK4A^* [25, 26]. Additionally, p53 is known to downregulate p16 through Id1-independent mechanisms [27].

## 3. Multiple Functions of p16

The p16 protein was discovered in the early 1990s, and was extensively studied for its ability to influence cell progression from G1 to S phase. p16 was shown to inhibit the kinase activities of the cyclin D-dependent kinases CDK4 [28] and CDK6 [29]. As cyclin D levels rise in G1, cyclin D binds the constitutively expressed CDK4 and CDK6 molecules. The resultant cyclin/CDK complexes phosphorylate pRB, leading to the release of active E2F that mediates transcriptional activation of a variety of proteins necessary for G1 to S progression and DNA replication, including cyclin E, cyclin A, and thymidine kinase [30]. Inhibition of pRB phosphorylation and E2F release in turn lead to inhibition of G1-S progression.

**Figure 1:**
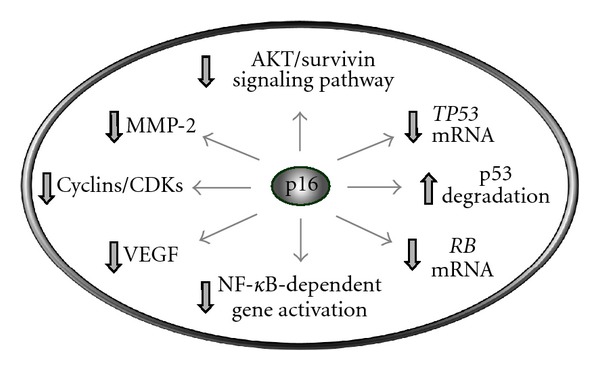
Multiple functions of the p16 tumor suppressor. This protein inhibits the kinase activities of CDK4 and CDK6 that mediate pRB phosphorylation [28–30], promotes MDM2-dependent degradation of p53 [40], downregulates AKT/survivin signaling [41], and represses the transcription of several genes including *RB* [39], *TP53* [40], *VEGF* (vascular endothelial growth factor) [13], *MMP-2* (matrix metalloproteinase 2) [32], and *NF-*κ*B* [38].

A number of additional biochemical and biological functions have since been documented for p16 (Figure 1). Numerous reports published in the late 1990s implicated a role for p16 in regulating angiogenesis [31], tumor invasion [32], cell spreading [33], and other fundamental cellular processes [34–37]. In part, p16 was shown to elicit such pleiotropic effects by modulating the expression or function of distinct target molecules, such as transcriptional downregulation of genes that encode vascular endothelial growth factor (VEGF) [31], matrix metalloproteinase 2 (MMP-2) [32], nuclear factor *κ*B (NF-*κ*B) [38], and pRB [39].

More recently, p16 was shown to negatively or positively regulate apoptotic cell death depending on the stimuli. Thus, p16 protected cells from undergoing apoptosis after DNA damage by downregulating the intrinsic-mitochondrial pathway [43], whereas it promoted the detachment-triggered apoptosis (a process called anoikis; Greek word for homeless) by downregulating AKT/survivin signaling [41].

In addition, a reciprocal relationship was demonstrated between p16 and p53, a key regulator of apoptosis. Huschtscha et al. [40], for example, reported that p16 regulates p53 expression by both decreasing *TP53 *transcription and increasing Mdm2-mediated p53 degradation. Functioning at the hub of the DNA damage surveillance network, p53 regulates many DNA-damage-triggered responses including transcription, DNA repair, cell-cycle checkpoints, apoptosis, and SIPS (reviewed in [6]). A reciprocal relationship between p16 and p53 was documented not only with cultured human and murine cells [27, 40], but also with human tumor xenografts [44] and with a transgenic mouse model that carries the entire human *p16^INK4A^* locus [45]. As extensively discussed by Rayess et al. [23], these and related studies established a critical role for p16 in cell-fate determination following genotoxic stress when p53 is inactivated. Thus, genotoxic stress (e.g., DNA damage, oncogenic RAS expression) triggers the increased generation of reactive oxygen species that activate the MAPK pathway, leading to MAPK-mediated p16 expression and p16-mediated responses (e.g., SIPS) in p53-deficient cells.

In short, p16 is a multifunctional tumor suppressor capable of forming a negative regulatory loop with p53 and influencing the expression of a large number of cancer-associated genes both directly (e.g., *RB*, *TP53*) and indirectly by inhibiting the transcription regulators NF-*κ*B and p53.

## 4. p16 Expression in Human Fibroblasts ****Undergoing Replicative Senescence

As mentioned earlier, evaluating the roles of p21 and p16 in different forms of senescence has been the subject of intensive research since their discovery in the 1990s. Initial studies suggested a sequential involvement of these proteins in replicative senescence of diploid human fibroblasts, with p21 activating cell-cycle arrest at the early stage of senescence, and p16 being crucial to maintain the senescent cell-cycle arrest [46, 47]. p16 was proposed to elicit growth arrest by inhibiting pRB phosphorylation, which results in sequestration and inhibition of the E2F family of transcription factors [1]. It was subsequently demonstrated that pRB is downregulated in cells undergoing senescence and becomes barely detectable in “late” senescent cells [47], suggesting that the long-term maintenance of the senescence phenotype can occur in the absence of pRB. Brookes et al. [48] observed considerable variability in the basal levels and kinetics of p16 accumulation in different human fibroblast strains. The levels of p16 increased with population doublings in two of the four normal fibroblast strains tested, with the other two stains showing little or no increase in p16 at late passages. These authors further demonstrated that p16-deficient human fibroblast strains are arrested at late passages and exhibit features of replicative senescence, and concluded that p16 might not be essential for the termination of fibroblast life span.

In 2007, two comprehensive review papers were published on the role of p16 in replicative senescence, with opposing conclusions. Thus, Cánepa et al. [49] suggest that p16 is a key regulator of replicative senescence and identified p16 as a molecular marker of this process, whereas Zhang [3] suggests that p16 is not related to replicative senescence mediated by telomere shortening, although the global expression of this CDK inhibitor might be increased in senescent cells. Examining the basis for such conflicting ideas is beyond the scope of the current paper. In what follows we will mainly focus on our own findings with human fibroblasts with different genetic backgrounds.

Most studies addressing the role of p16 in telomere-directed senescence relied on the evaluation of global p16 protein levels. However, such measurements can be misleading because not all proteins are uniformly expressed among cells within a putatively “clonal” population. Indeed, Herbig et al. [2] employed single-cell evaluation techniques and demonstrated that p16 protein levels can be heterogeneous among cells within a given senescent culture, with the majority of cells exhibiting undetectable levels of p16, and only a small proportion containing extremely high levels. We have reported similar observations with human normal and ataxia telangiectasia (AT) fibroblast strains that express wild-type p53 [42]. Normal and AT fibroblast cultures entered the state of senescence after approximately 70 and 50 population doublings, respectively. The majority (>90%) of cells undergoing replicative senescence exhibited strong nuclear accumulation of p21, but did not express p16 [42].

A different scenario was apparent in Li-Fraumeni syndrome (LFS) fibroblast strains. The LFS strains studied by us are heterozygous for *TP53* mutations at either codon 254 (strains 2675T and 2674T) or codon 234 (strain 2800T). Such mutations result in either compromise (codon 254) or absence (codon 234) of p53-dependent transcription, as evident from the ability of the cells to upregulate *p21^WAF1^* mRNA and p21 protein in response to DNA damage [50, 51]. Given that the p53-p21 pathway is a key mediator of senescence [6], it was of interest to determine the fate of LFS cells as a function of culture age. Vaziri et al. [52] reported that strains 2674T and 2675T lose the wild-type *TP53* allele at late passages and (surprisingly) undergo replicative senescence. We demonstrated that all three LFS stains undergo replicative senescence after ~80 (2800T), ~90 (2675T), and ~100 (2674T) population doublings and that such cells fail to express p21 but express very high levels of p16 [42]. Early-passage cultures of these LFS strains do not express p21 or p16.

**Figure 2:**
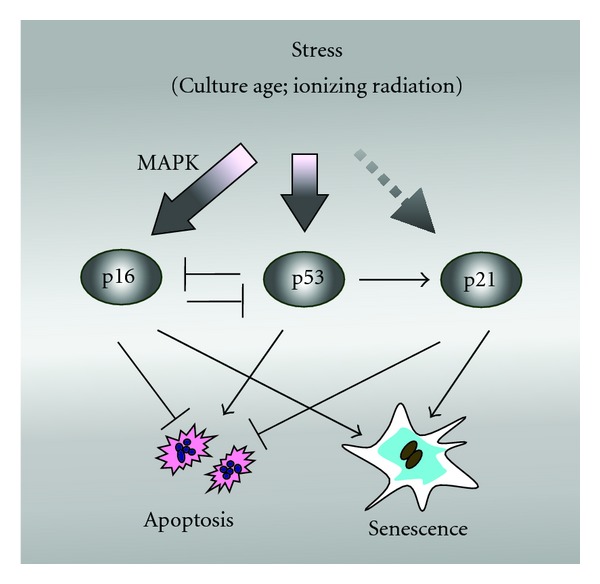
Model illustrating the involvement of the p16, p53, and p21 tumor suppressors in senescence of human fibroblast cultures [42]. In p53-proficient (normal) fibroblasts, telomerase shortening (e.g., as a function of culture age) or exposure to DNA-damaging agents results in activation of p53, which represses p16 and transcriptionally activates p21. The latter protein suppresses apoptosis and triggers senescence. On the other hand, p53-deficient (Li-Fraumeni syndrome) fibroblasts respond to stress by upregulating p16 which suppresses apoptosis and triggers senescence.

Collectively, these results led us to propose the model presented in Figure 2, in which p16 functions in a redundant pathway of replicative senescence in human fibroblasts, triggering this process only in the absence of wild-type p53 activity. This model is consistent with the aforementioned recent discoveries demonstrating a negative interrelationship between p16 and p53.

## 5. p16 Expression in Human Fibroblasts ****Undergoing SIPS

In our previous work, ionizing radiation exposure triggered extensive SIPS but marginal (if any) apoptosis in early-passage cultures of p53-proficient (normal and AT) and p53-deficient (LFS) fibroblasts [42]. The proportion of cells undergoing SIPS after irradiation correlated with the proportion of cells expressing p21 but not p16 in p53-proficient cultures, and with the proportion of cells expressing p16 but not p21 in p53-deficient (strain 2800T) cultures. These observations are consistent with the properties of p21 [6] and p16 (see above), both of which are known to downregulate the intrinsic-mitochondrial pathway of apoptosis and to induce senescence. It is important to note that a small proportion (<5%) of cells within cultures of normal and AT strains did express p16 before radiation exposure which remained virtually unchanged after irradiation.

In an earlier work, we also determined the relationship between SIPS and expression of p21 and p16 in normal human fibroblasts exposed to ultraviolet light (UV). UV exposure triggered extensive SIPS which was associated with sustained nuclear accumulation of p21 [53]. Normal fibroblasts did not express p16 before and after exposure to UV [53].

These observations provide further support for our model (Figure 2) in which p16 and p53/p21 function in non-overlapping pathways of senescence.

## 6. p16 Expression in Human Cancer ****Cells Undergoing SIPS

In 1994, several reports demonstrated that the majority (~85%) of human cancer cell lines do not express p16 due to deletion, mutation, or silencing of the *INK4A *locus [54–56]. This discovery led to the notion that such cancer cells might not undergo growth arrest through the process of senescence. In 1999, however, Chang et al. [57] reported the induction of SIPS in p16-null and p53 wild-type human cancer cells (e.g., HT1080 fibrosarcoma) after exposure to different genotoxic agents, including ionizing radiation. Numerous reports have since demonstrated the induction of SIPS by ionizing radiation and chemotherapeutic agents in different solid tumor-derived cell lines that express wild-type p53. Among the cell lines studied by us, HCT116, A172, and SKNSH showed a response to ionizing radiation similar to normal human fibroblasts in terms of clonogenic survival, SIPS, and p21 expression [58]. As seen with normal fibroblasts, these cancer cell lines did not express p16 before and at different times (between 24 and 96 h) after ionizing radiation exposure (unpublished observation cited in [58]). Lack of p16 expression in HCT116 cells is consistent with the presence of a frameshift mutation in one allele of the *INK4A* gene, and hypermethylation of the promoter of the other allele [59]. Like HCT116, the widely used cancer cell lines A549 and MCF7 that undergo SIPS after DNA damage express wild-type p53 but do not express p16 [60–62].

These observations, together with our findings with human normal and LFS fibroblasts suggesting the involvement of p16 in a redundant pathway of senescence [42], prompted Wang and associates [62] to test whether this model is also applicable to solid tumor-derived cells. Ionizing radiation exposure triggered SIPS not only in a p53-wild-type and p16-deficient cell line (A549 lung carcinoma), but also in two p16-proficient and p53-mutated cell lines (ABC-1 adenocarcinoma and HCC44 lung carcinoma). Immunofluorescence analysis revealed that the induction of SIPS in these p16-proficient and -deficient cancer cell lines was associated with nuclear accumulation of p16 and p21, respectively. Consistent with our findings with LFS fibroblasts [42], the induction of p16 in ABC-1 and HCC44 cells was observed within days following 8 Gy irradiation [62].

**Figure 3:**
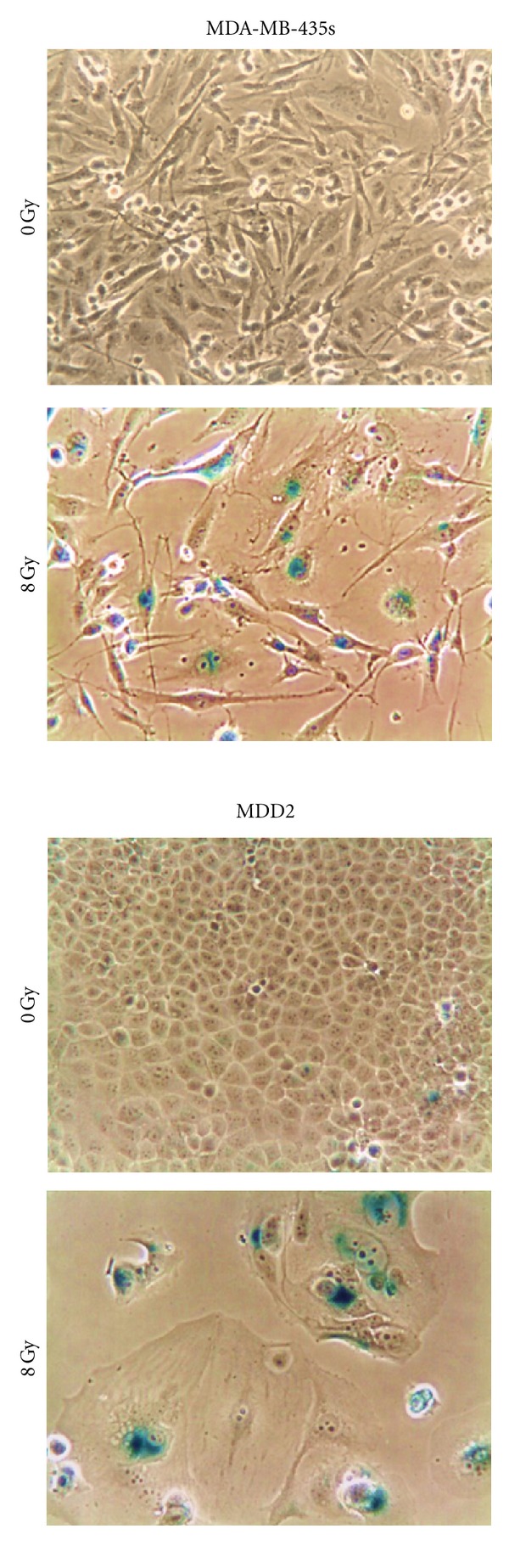
Phase-contrast photomicrographs showing SIPS in breast cancer cell lines that express mutant p53. Cells were exposed to ^60^Co *γ*-radiation (8 Gy) or sham-irradiated (0 Gy), incubated for seven days, and evaluated for features of SIPS (flattened and enlarged cellular morphology and positive (blue) staining in the senescence-associated *β*-galactosidase assay).

**Figure 4:**
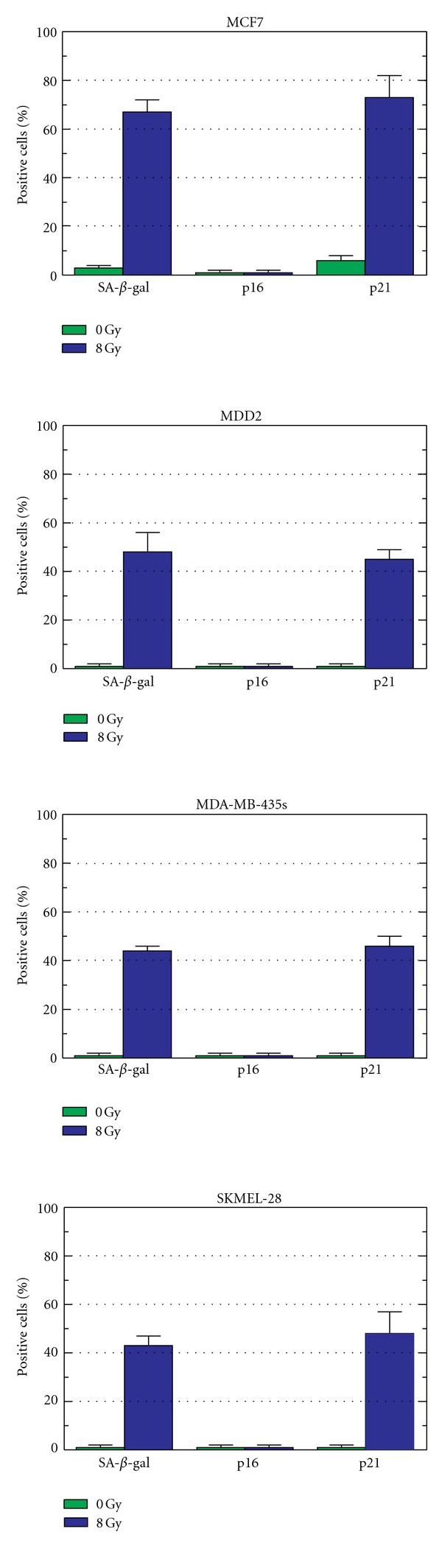
Relationship between the proportion of senescence-associated *β*-galactosidase (SA-*β*-gal)-positive cells, p16-expressing cells, and p21-expressing cells before and seven days after exposure to *γ*-radiation (8 Gy). Bars, SE.

We extended these studies to the mutant p53-expressing cell lines MDA-MB-435s, SKMEL-28, and MDD2; the MCF7 cell line was also evaluated as a control. Both MDA-MB-435s and SKMEL-28 cell lines lack wild-type p53 activity due to *TP53* mutations, but do express p16 [63–65]. The MDD2 cell line is a variant derived from MCF7 by transfection with a dominant negative mutant of p53 [66]. Unlike MCF7, MDD2 cells lack wild-type p53 activity [66, 67]. The results for seven days after irradiation are presented in Figures 3 and 4. As expected, ionizing radiation (8 Gy) triggered SIPS in p53 wild-type cells (MCF7), which correlated with expression of p21 but not of p16. Irradiation of mutant p53-expressing cell lines also triggered SIPS and, surprisingly, this response was associated with induction of p21 but not of p16.

In short, the results discussed above clearly demonstrate that DNA damage can trigger SIPS in human cancer cell lines expressing wild-type or mutant p53, and that this response is associated with nuclear accumulation of p21 in the majority of cases, and with induction of p16 in some cases. Further studies are warranted to determine the basis for the nuclear accumulation of p21 at late times (7 days) after irradiation in some cancer cell lines that express mutant p53 and to elucidate the reason why some p53-mutated cell lines (e.g., MDA-MB-453 s and SKMEL-24, but not ABC-1 and HCC44) exhibit the delayed nuclear accumulation of p21 after irradiation but not of p16. It is noteworthy that p21 functions as a repressor in p53-mediated downregulation of genes such as *BCL-2*, *MCL-1*, *survivin*, and *MDR-1* (reviewed in [6]). As a working model, we propose that p21 might also be responsible for repressing p16, which might explain why some p16-proficient cell lines that exhibit nuclear accumulation of p21 after DNA damage do not concomitantly exhibit nuclear accumulation of p16.

## 7. Conclusion

Since their discovery in the 1990s, the CDK inhibitors p21 [6] and p16 (Figures 1 and 2) have been shown to exhibit a variety of biochemical and biological functions, most of which are independent of their major influence on pRB-regulated G1-S progression. Herein, we have highlighted the growing diversity of p16 functions, examined recent studies that implicate a role for p16 in a redundant pathway of senescence that operates in cells lacking wild-type p53, and reported new data demonstrating the complexity of DNA damage-induced SIPS in human solid tumor-derived cell lines expressing mutant p53.

At first glance, the findings discussed above may appear contradictory to several reports which suggested p16-directed senescence induced by different stimuli in p53 wild-type (e.g., normal) human cells, including SIPS triggered by DNA-damaging agents (e.g., bleomycin) (reviewed in [3]). However, it is important to note that the majority of studies that did not support a role for p16 in SIPS used single-cell measurements at 7 days (this report) or shorter times [42, 53, 62] after exposure, whereas most studies that did support a relationship between p16 expression and SIPS used global protein measurements and observed significant p16 induction at much longer times (e.g., 30 days) after introduction of DNA damage (e.g., [68]). Thus, it is reasonable to conclude that p16 might be dispensable for activation and relatively short-term (several days) maintenance of SIPS in p53 wild-type cells.

An open question remains: is p16 required for the long-term maintenance of the SIPS response in cells expressing wild-type p53? Addressing this question is of particular importance in the context of cancer therapy in view of a large body of recent evidence demonstrating that a proportion of cells undergoing SIPS after DNA damage can eventually escape this response, giving rise to aneuploid offspring exhibiting highly metastatic and therapy-resistant properties (reviewed in [5–7]).
